# Hülle Cells of Aspergillus nidulans with Nuclear Storage and Developmental Backup Functions Are Reminiscent of Multipotent Stem Cells

**DOI:** 10.1128/mBio.01673-20

**Published:** 2020-08-11

**Authors:** Danielle M. Troppens, Anna M. Köhler, Rabea Schlüter, Michael Hoppert, Jennifer Gerke, Gerhard H. Braus

**Affiliations:** a Department of Molecular Microbiology and Genetics, Georg-August-University, Göttingen, Germany; b Imaging Center of the Department of Biology, University of Greifswald, Greifswald, Germany; c Department of General Microbiology, Georg-August-University, Göttingen, Germany; Universidad de Córdoba

**Keywords:** sexual development, Hülle cell development, fungal stem cell, fungal biology, filamentous fungi, life cycle, fungal development, Hülle cell germination

## Abstract

Some aspergilli are among the most cosmopolitan and ecologically dominant fungal species. One pillar of their success is their complex life cycle, which creates specialized cell types for versatile dispersal and regenesis. One of these cell types is unique to aspergilli—the Hülle cells. Despite being known for over a century, the biological and ecological roles of Hülle cells remain largely speculative. Previously reported data on *in vivo* Hülle cell formation and localization have been conflicting. Our quantification reveals that Hülle cells can occur at all locations on hyphae and that they show cellular activity similar to that seen with adjacent hyphae, indicating that they develop as intricate parts of hyphal tissue. In addition, we show that during sexual development associated with two parental strains, the typically multinucleate Hülle cells can inherit nuclei from both parents, indicating that they may serve as genetic backups. We provide an easy, reproducible method to study Hülle cell biology and germination with which we investigate the 90-year-old puzzle of whether and how Hülle cells germinate. We present clear evidence for the germination of Hülle cells, and we show that Hülle cells grow hyphae that develop into a spore-producing colony. Finally, we show that Hülle cell-derived colonies produce conidiospores faster than spore-derived colonies, providing evidence for an as-yet-undescribed developmental shortcut program in Aspergillus nidulans. We propose that Hülle cells represent a unique cell type as specialized hypha-derived sexual tissue with a nucleus storage function and may act as fungal backup stem cells under highly destructive conditions.

## Abstract (precis)

The *in vivo* identification of Hülle cells in cases of aspergillosis infections in animals and humans illustrates their biological relevance and suggests that they might be involved in pathogenicity. It is striking that aspergilli have developed and maintained a multinucleate nurse cell that is presumably energy-intensive to produce and is usually found only in higher eukaryotes. Our findings shed light on how the understudied Hülle cells might contribute to the success of aspergilli by acting not only as nurse cells under detrimental conditions (sexual development) but also as fungal backup stem cells with the capacity to produce genetically diverse spores in an accelerated manner, thereby substantially contributing to survival in response to predator attack or under otherwise severely destructive conditions. Our study solved the 90-year-old puzzle of Hülle cell germination and provides easy, reproducible methods that will facilitate future studies on biological and ecological roles of Hülle cells in aspergilli.

## INTRODUCTION

Fungi and their fungal ancestors have inhabited this planet for about one billion years (1), granting them plenty of time to evolve and adjust to specific niches. The success of one such group of fungi, the aspergilli, is impressively illustrated by their abundance; they are found in virtually all terrestrial habitats, both natural and man-made (2–6). Aspergilli are usually considered compost dwellers; however, they have been recognized as opportunistic pathogens of plants, animals, and humans. One pillar of their success is the efficient and, for some species, versatile form of spore dispersal that is inherent in their complex life cycle.

The life cycle of the model fungus Aspergillus nidulans is relatively well understood. Asexual development induced by exposure to light and normal oxygen levels yields a radially expanding colony that develops asexual conidiospores containing a single nucleus (7, 8). Darkness and low-oxygen conditions induce sexual development that produces a fruiting body containing sexual binucleate ascospores (9–11). Two nuclei are responsible for the formation of all ascospores within one cleistothecium (12), which is why the presence of two different nuclei in ascospores of the same cleistothecium is the result of an out-crossing, i.e., the fusion of two genetically different strains (13).

Sexual development in A. nidulans features cells of one cell type that seem to be uniquely associated with aspergilli—the Hülle cells, thus named after their initial identification as an envelope of bubbles (in German: “Blasenhülle”) (14). Despite the early discovery of Hülle cells, it still remains largely speculative what advantage the assumingly energy-consuming production of Hülle cells confers or how this cell type contributes to the success of aspergilli. Previous studies providing evidence of cellular and enzymatic activity in Hülle cells suggested that they may actively support and protect the developing fruiting body (15–18). Expression of a mutanase (19) and exhibition of chitin synthase activity (20, 21) suggest that Hülle cells modulate cell wall (CW) components. In accordance with the nursing hypothesis, we previously reported that an A. nidulans strain lacking the developmental regulator LaeA produced significantly fewer Hülle cells. Cleistothecia, though containing mature ascospores, remained small, suggesting that they were malnourished as a consequence of the lack of Hülle cells (22).

Ellis and coworkers first described the multinucleate state of Hülle cells in an ultrastructural analysis (15), and the description was corroborated by Carvalho et al. (23). Despite the discovery of the multinucleate state of Hülle cells, the distribution of nuclei into Hülle cells in a heterokaryon remained unstudied.

A number of studies mentioned the ability of Hülle cells to germinate (15, 23–25); however, detailed information on this process was missing, presumably due to the difficulty of studying this cell type and to the lack of a robust and reproducible method. The fate of supposedly germinated Hülle cells remained entirely unknown. Such knowledge is, however, crucial for understanding their role within the life cycle.

The overall aim of our study was to provide a comprehensive idea of the biology and function of Hülle cells in A. nidulans and to uncover their developmental potential by investigating (i) whether Hülle cells act as separate entities or as part of the hyphal tissue *in vivo*, (ii) which parental nuclei are distributed into Hülle cells in a crossing event, and (iii) how Hülle cells respond and develop when detached from the hyphal network.

## RESULTS

### Hülle cells are evenly distributed as an active part of the hyphal network.

**FIG 1:**
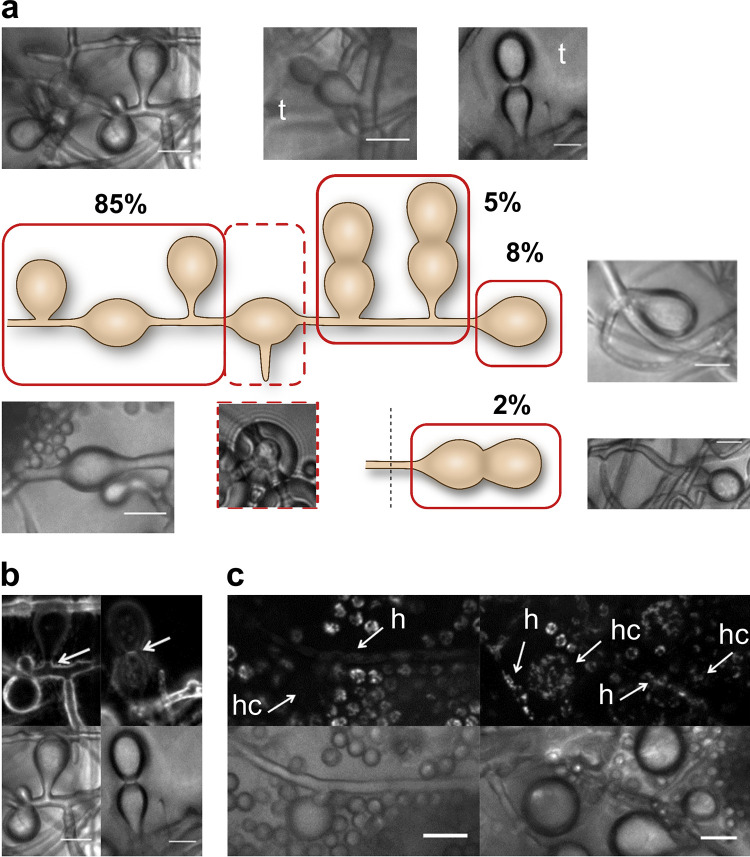
Formation, septation, and cellular activity of Hülle cells of A. nidulans. (a) The majority (85%) of Hülle cells are formed as single Hülle cells along the hyphal cortex or within the hyphae. A minority (10%) are formed at the hyphal tip. A similarly small amount (7%) of the cells occur as tandem Hülle cells (t). Hülle cells might also be formed at hyphal branching sites as suggested by the presence of detached Hülle cells with three openings (dotted box and dotted image). Data represent averages of results from three biological replicates (*n* > 150). The standard deviations determined for intercalary position (90%) and apical position (10%) are 2.65 and 2.65. The schematic does not represent a typical hypha but instead shows the locations of Hülle cells found on separate hyphae. (b) Septa were formed at the Hülle cell opening and between tandem Hülle cells. (c) Cellular activity, visualized by mitochondrial fluorescence, of Hülle cells (hc) was similar to that of neighboring hyphae (h) with low activity (left) and high activity (right). Scale bar = 10 μm.

Hülle cells were previously described as emerging either from hyphal tips or at an intercalary position; however, studies did not consistently describe the two types (14, 15, 25–27). To further characterize Hülle cell biology and function, we systematically analyzed Hülle cell formation by quantifying sites of Hülle cell emergence at hyphae of A. nidulans. The majority (90%) of Hülle cells were formed by branching off the hyphal cortex (with or without a stalk) or presumably by swelling of hyphal segments. A smaller proportion (10%) developed at the hyphal tip (Fig. 1a). Separated Hülle cells with three visible openings at original attachment sites suggested that formation also occurs at filament branching sites (Fig. 1a, dotted image). Most (93%) Hülle cells from the colony edge emerged as single cells and only a few (7%) as tandem Hülle cells (which are thus far undescribed), among which 22% (2% of the total number of Hülle cells) arose at tips (Fig. 1a).

Septation plays a crucial role in the development of heterogeneous multicellular fungi (28, 29) and was observed at Hülle cell attachment sites, between tandem cells (Fig. 1b), or at both sides of internal Hülle cells. Separation of hyphae from Hülle cells by septa suggests that Hülle cells form discrete compartments for certain cellular processes.

Hülle cells are thought to be involved in the development of the fruiting body as nursing cells (22). We used a strain with a fusion gene encoding green fluorescent protein (GFP) and the N-terminal part of mitochondrial citrate synthase (N-cit-1), including the mitochondrial import signal sequence (30). The mitochondria can be visualized in Hülle cells only if cellular activity associated with mitochondrial import *in vivo* is functional during fruiting body development. We found that Hülle cell activity was usually similar to the activity seen in the adjacent hypha. Thus, Hülle cells showed little or no fluorescence when there was low fluorescence in the attached hypha and a high level of fluorescence when there was a high level of fluorescence in the attached hypha (Fig. 1c).

Our quantifications show that sexual hyphae of A. nidulans produce Hülle cells at any available location in the vicinity of the cleistothecium, thus establishing an even distribution throughout the hyphal network surrounding the developing fruiting body. This suggests that the fungus has the potential to use all the available space to produce the visible and presumably protective envelope around the fruiting body by the use of Hülle cells. Taken together with the finding that, even as separate compartments, Hülle cells communicate with and represent an active part of the hyphal network, these results support the idea of the involvement of auxiliary cells.

### Hülle cells can acquire nuclei of two distinct parental strains during the sexual cycle.

Hülle cells represent a hallmark of sexual development, and so far, they have been investigated only in self-fused structures (homokaryon). We crossed two A. nidulans strains that carried N-terminally red fluorescent protein (RFP)-tagged and GFP-tagged histones, respectively, to investigate which nuclei would be distributed to Hülle cells when two different strains are fused (heterokaryon).

**FIG 2:**
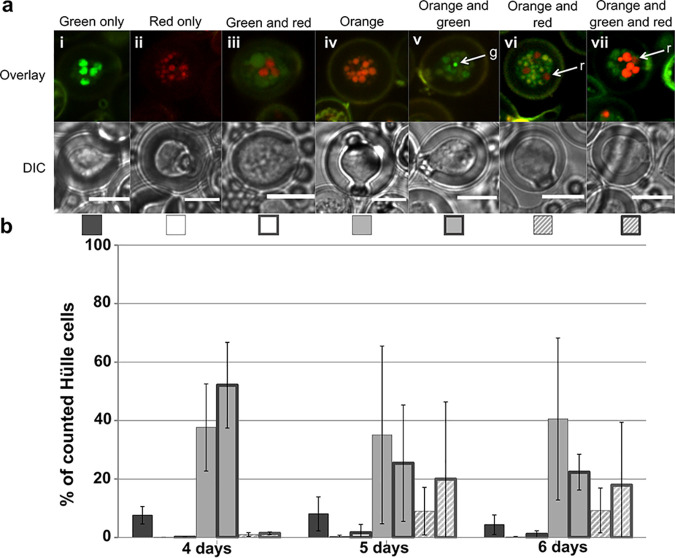
Nuclear distribution in Hülle cells of a heterokaryon. (a) Hülle cells developed during crossing of two strains carrying either RFP-tagged or GFP-tagged histones contained nuclei showing only green fluorescence (i), only red fluorescence (ii), both green fluorescence and red fluorescence (iii), only orange fluorescence (iv), a mixture of orange fluorescence and green fluorescence (v), or a mixture of orange fluorescence and green fluorescence and red fluorescence (vi). Arrows indicate the less abundant single-colored nuclei (g = green, r = red). GFP and RFP signals were imaged individually, and an overlay was created. Scale bar = 10 μm. DIC, differential interference contrast. (b) The majority of Hülle cells contained nuclei that showed green and red fluorescence (orange) or orange fluorescence in combination with nuclei emitting only green fluorescence (orange plus green), whereas Hülle cells with nuclei emitting orange fluorescence in combination with red fluorescence were rare, as were all other combinations with nuclei emitting red fluorescence. Variations in all variants increased after 5 and 6 days. Data represent averages of results from three biological replicates (*n* > 120), with standard deviations indicated as error bars. Orange fluorescence might not have been visually recognizable as such when one color dominated the other.

Hülle cells containing nuclei emitting only green or only red fluorescence (either alone or in combination) made up less than 10% of all Hülle cells counted over time (Fig. 2a, panels i to iii; see also Fig. 2b). The majority of nuclei emitted both red and green fluorescence signals, here referred to as orange, which can be explained by the presence of newly synthesized histones of different nuclei packed into the same new nucleus (Fig. 2a, panel iv). We also found these orange nuclei in combinations with the less abundant red and green nuclei that emitted only a single fluorescence signal (Fig. 2a, panel v to vii). Orange nuclei combined with or not combined with single fluorescent nuclei were found in more than 90% of all counted Hülle cells over time (Fig. 2b).

Our results show that both parental nuclei can be distributed to Hülle cells of a heterokaryon, suggesting that Hülle cells are mostly formed by the heterokaryotic mycelium and that Hülle cells might serve as a nuclear reservoir or backup system.

### Hülle cells regain cellular activity and develop new hyphae when detached from hyphal mycelium.

**FIG 3:**
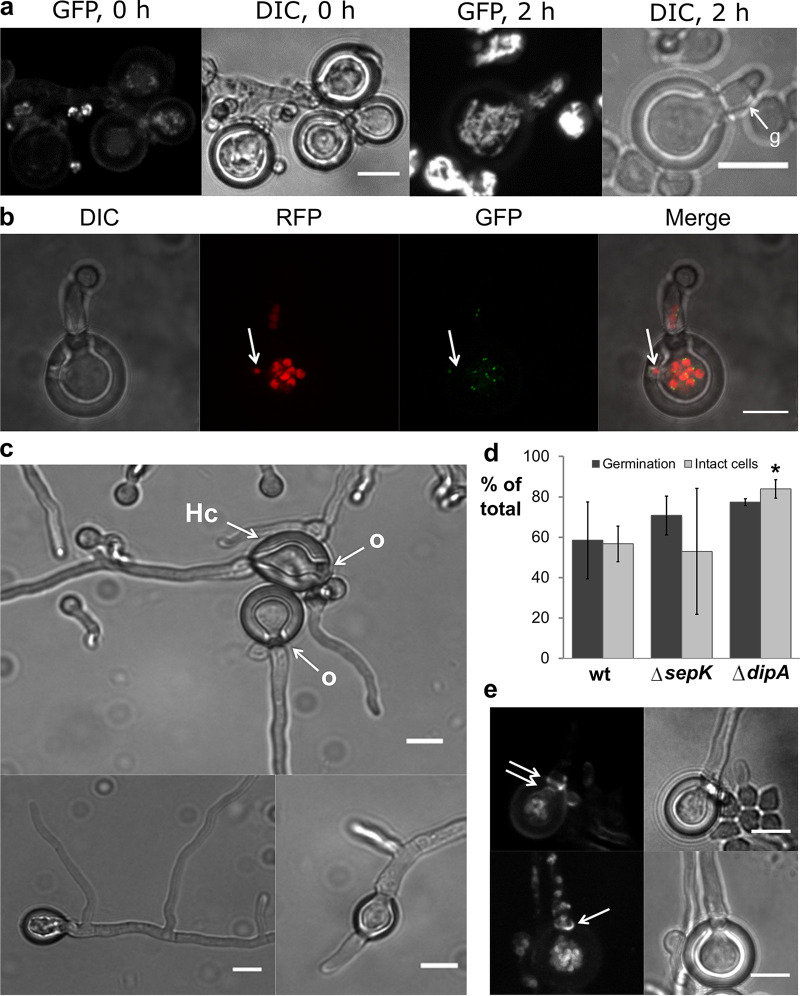
Mitochondrial activity, localization of GFP-SepK, and hyphal growth in detached Hülle cells of A. nidulans. (a) Immediately after detachment from the mycelium (0 h), Hülle cells showed weak punctuated mitochondrial fluorescence (left panels). After a minimum of 2 h on fresh medium (2 h), some of the Hülle cells showed a tubular mitochondrial network that extended into a newly developed presumed germ tube (g) (right panels). (b) Dots of fluorescence indicative of GFP-SepK expression were visible after an incubation of 4 h at or near nuclei within the Hülle cell as well as in a newly formed germ tube and in a new germination site (arrow). Scale bar = 10 μm. (c) Hülle cells (Hc) had developed hyphae that emerged from openings 7 h after detachment (o). Hülle cell-derived hyphae were able to branch (bottom left). Hülle cells containing two openings were able to grow hyphae from both (bottom right). Scale bar = 10 μm. (d) An average of 59% of the detached Hülle cells germinated in the wild-type strain. The rates of germination were similar in the *ΔsepK* and *ΔdipA* septation mutants. Data represent results from three independent biological replicates, with a minimum of 200 Hülle cells counted per replicate and strain. An average of 57% of detached Hülle cells were unstained by propidium iodide and therefore are considered representative of intact cells. This rate is similar to the germination rates seen in wild-type and septation mutants. The rate of intact cells in the *ΔsepK* mutant was similar to wild-type rates, whereas *ΔdipA* Hülle cells were more viable than wild-type ones (Student’s *t* test; *, *P* < 0.01). Data represent averages of results from three independent biological replicates (*n* > 140), with standard deviations indicated as error bars. Scale bar = 10 μm. (e) Two septa were seen in growing Hülle cells 7 h after detachment (two arrows). In some cases, only one septum is visible. The outer septum appeared as a septal ring (one arrow). Septa were stained using FM4-64. Scale bar = 10 μm.

On the basis of the finding that Hülle cells can contain nuclei from two parents, we investigated the developmental potential of Hülle cells. Directly after detachment from the mycelium (see detailed description in Materials and Methods), Hülle cells of a strain with fluorescently labeled mitochondria showed only weak fluorescence or no fluorescence, indicating that the cells were inactive at that time. In contrast, spores in their vicinity showed fluorescent mitochondria (Fig. 3a, left panels). However, after incubation for at least 2 h on fresh medium, some of the detached Hülle cells showed fluorescent mitochondrial networks, suggesting a revival of cellular activity. These mitochondrial networks were visible mainly within the Hülle cell but also extended into newly developed protrusions (Fig. 3a, right panels). These observations demonstrate that after separation from the mycelium, at least a subpopulation of Hülle cells regained cellular activity when provided with nutrients.

In A. nidulans, SepK is a homolog of a component of the septation initiation network that was described for the fission yeast Schizosaccharomyces pombe and is involved in mitosis-dependent septum formation (31, 32). SepK colocalizes to the spindle pole body during mitosis in A. nidulans (33) and is therefore an indicator for nuclear division and cytokinesis. We examined the expression level of the GFP-labeled SepK protein in a strain additionally containing RFP-labeled nuclei. Detached Hülle cells showed dot-like fluorescence signals 4 h after detachment, indicating expression of the GFP-SepK fusion protein (Fig. 3b). The signals were always found at or near a nucleus within the Hülle cell, in newly formed (presumed) germ tubes, or at potential new germination sites (Fig. 3b, arrow). Our microscopic data suggest that the depicted cell was currently undergoing or preparing for mitosis and that Hülle cells therefore bear the potential for nuclear division and migration.

Subsequently, we further monitored the growth of detached Hülle cells. Seven hours after detachment, the presumed germ tubes described above were found to have indeed elongated to form hyphae that always emerged from openings (o) (Fig. 3c). These Hülle cell-derived hyphae formed one or more branches (Fig. 3c, bottom left) and developed from either one or two openings (Fig. 3c, top and bottom right).

An average of 59% of Hülle cells (*n* > 200) developed hyphae within 7 h after detachment in the wild-type strain (Fig. 3e). One possible explanation for the remaining 41% not growing is that they lost cell viability due to mechanical damage during detachment. Thus, the cell viability of detached Hülle cells was examined. An average of 57% of the Hülle cells remained unstained after propidium iodide treatment (*n* > 140) (Fig. 3d). Since this rate is similar to the germination rate, it is likely that cell viability accounts for the number of Hülle cells retaining their ability to grow.

The most vulnerable sites that might be responsible for loss of cellular integrity are the Hülle cell openings. It was previously reported that Hülle cells show two septa at such locations (15). We confirmed this finding (Fig. 3e) but did not always observe two septa. Fluorescent staining also suggests that the outer septum is in fact a septal ring (Fig. 3e, left panel) as it is found at budding sites in the budding yeast Saccharomyces cerevisiae (34). The positioning, the number, and the structure of the septa might play important roles in maintaining cellular integrity and viability in Hülle cells. Therefore, we investigated hyphal growth in septation mutants. The Δ*sepK* mutant has been described previously as being deficient in conidiospore formation and showing a delay in septation (33). Another septation mutant, the Δ*dipA* strain, was previously found to have an increased frequency of septation (35), which might have an effect on the stability of hyphae and Hülle cells when damage occurs.

The germination rate did not significantly change between the *ΔsepK* and *ΔdipA* septation mutants and the wild-type strain (Fig. 3d). This indicates that neither delayed septation nor increased septation frequency reduced the ability of Hülle cells to germinate. We provide clear evidence for Hülle cell germination and demonstrate that it can be initiated from more than one site but only from Hülle cell openings.

### Hülle cell-derived hyphae form conidiophores and mature conidiospores.

**FIG 4:**
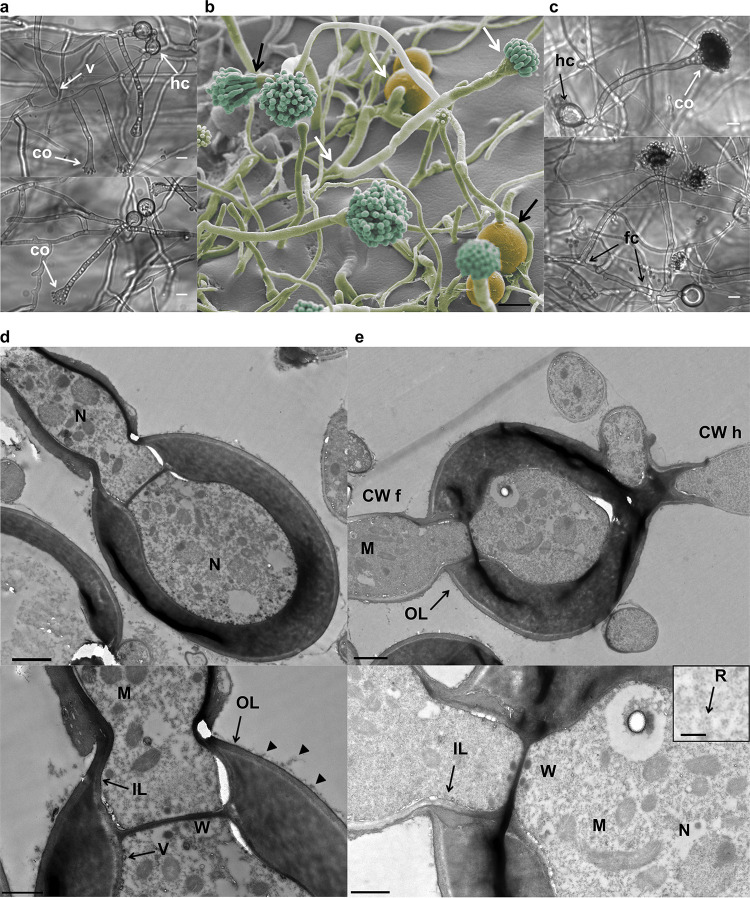
Development of conidiophores and conidia derived from Hülle cells. (a) Hülle cells (hc) develop vesicles (v) and, subsequently, young conidiophores (co). Hülle cell-derived conidiophores are mostly formed from hyphal branches (top) and can be directly formed from the Hülle cell (bottom). (b) Scanning electron micrograph of germinated Hülle cells with one conidiophore developed from a branch (white arrows) and one developed directly (black arrows). (c) Hülle cells develop mature conidiophores (co) that arise from a foot cell (fc) with the typically thicker cell wall than normal hyphae. Scale bars (a to c) = 10 μm. (d) Transmission electron micrographs of a germinated and highly active Hülle cell (hypha extended into a conidiophore that was outside the current view) with nuclei (N) (top), mitochondria (M), and Woronin bodies (W) at the septum (magnified inset image, bottom). The Hülle cell shows distinct layers outside the cell wall (OL) and on the inner side (IL), where transport vesicles (V) are frequently located. Additional structural elements are located at the outside layer (arrowheads). Note the thick wall of the Hülle cell. (e) Transmission electron micrographs of an active Hülle cell with three germination sites. The thicker cell wall of the left hypha presumably represents a foot cell (CW f) compared to the thinner cell wall of the right hypha (CW h). The cell contains a nucleus (N), elongated and spherical mitochondria (M), numerous ribosomes (R) (magnified inset image, bottom), and Woronin bodies (W) at the septum. Scale bars (d to e) = 2 μm (top), 1 μm (bottom), and 0.25 μm (magnified inset).

Growth of Hülle cells was further monitored for up to 20 h after detachment. Hyphae that derived from Hülle cells developed conidiophores that resembled normal A. nidulans conidiophores (Fig. 4a). Vesicles (v) and young conidiophores (c) started to emerge from hyphae of individual Hülle cells after an incubation period of about 12 h. These mostly originated from branches but in rare cases also emerged directly from the Hülle cell opening (Fig. 4a and b). Hülle cell-derived hyphae can develop conidiophores at early branches or later branches or both (Fig. 4a). After incubation for about 14 to 16 h, conidiophores carried the first conidiospores (Fig. 4b and c). As described previously for spore-derived asexual development (8), Hülle cell-derived conidiophores originated from foot cells (fc) characterized by their thicker cell wall (Fig. 4c).

Transmission electron micrographs of germinated Hülle cells show that they were highly active during the process of conidiophore formation, which is illustrated by the large amount of ribosomes (R) and mitochondria (M) present (Fig. 4d and e). Typically, one or several nuclei (N) were visible in the Hülle cell and the developing hyphae (Fig. 4d and e). An internal septum that was surrounded by Woronin bodies (W) was frequently present (Fig. 4d and e). We did not observe the basal septum that was described in a previous report in maturing Hülle cells (15); however, depending on where the Hülle cell was cut, the cell wall was still visible at the germination sites (Fig. 4e). We propose that the cell wall at this location continues to form an opening that corresponds to the septal ring that we had observed by fluorescence microscopy (Fig. 3d). The thick cell wall was surrounded by a thin outer layer (OL), on which additional structural elements were visible (arrowheads) (Fig. 4d and e). These structures might represent exopolysaccharides. A similar but slightly thinner inner layer (IL) was present at the inner side of the cell wall where vesicles (V) that might be involved in transport into the cell wall were frequently observed. This inner layer seemed to extend into the growing hypha, whereas the outer layer seemed to be exclusive to the Hülle cell (Fig. 4d and e). The cell wall itself appeared delicate in its structure; a visual effect that was caused by the creases. However, these creases might simply represent an artifact of the sectioning procedure due to incomplete infiltration of the cell wall by the resin. This suggests a high structural density of the Hülle cell wall. It is noticeable that the thickness of the cell wall in the developing hyphae varied (Fig. 4e). As conidiophores in some cases emerged directly from the Hülle cell opening, it can be concluded that the thicker cell wall is indicative of a foot cell (CW f), whereas the thinner cell wall points to a normally grown hypha (CW h).

We show here that Hülle cells expand the fungal developmental program potential by actively forming asexual conidia that can be dispersed through the air.

### Hülle cells accelerate conidiospore formation and undergo the normal life cycle.

Hülle cells and conidiospores were isolated onto separate plates to directly compare the timings of conidiophore development. Whereas transfer of a single isolated Hülle cell by the use of a micromanipulator onto a new plate was difficult and resulted in germination in only 2 of 100 cases, isolation of a group of 20 Hülle cells onto a new plate increased the probability that a colony would form to approximately 50%. Isolating 20 conidiospores in the same way always yielded a colony. We quantified the germination rate of Hülle cells in comparison to that of conidiospores to determine how many cells truly contributed to a successful colony. We distinguished between clear germination and potential germination, with putative germination sites facing downwards (see Materials and Methods). In 13 quantified colonies originating from 20 Hülle cells, fewer than 10 of the 20 Hülle cells (<50%) always potentially germinated and fewer than 5 Hülle cells (<25%) clearly germinated. In all 19 quantified conidiospore-derived colonies, all of the visible spores (100%) always germinated.

**FIG 5:**
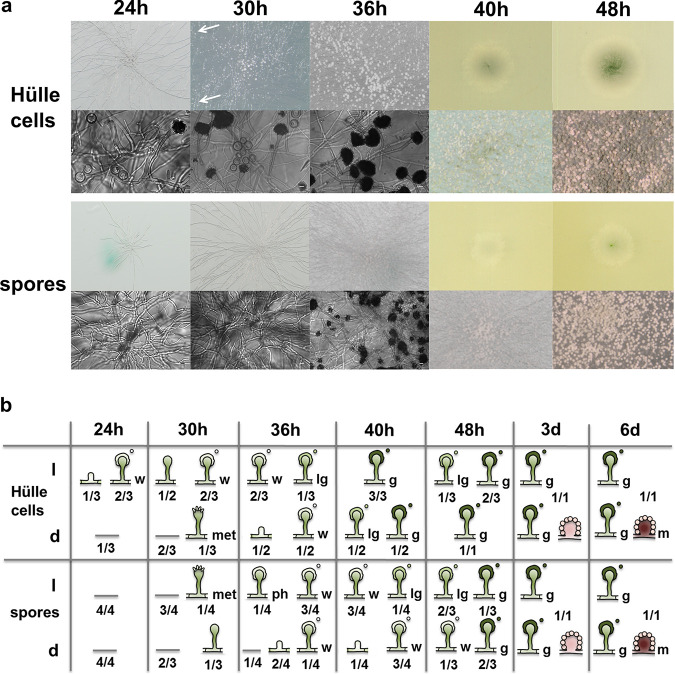
Hülle cells form conidiophores in an accelerated manner. (a) Representative images of asexual structures found for Hülle cells or conidiospores grown under asexual conditions for time points 24 to 48 h. If the appearances of the colonies differed between biological replicates, the colony showing the least extreme appearance (representing the average) was selected for display. Arrows indicate outliers of conidiophores formed outside the colony center. (b) From each of at least three biological replicates, the most highly developed cellular structure derived from Hülle cells or spores during asexual (l) and sexual (d) development is represented together with its frequency (1/3). The symbols represent these structures, including stalk, vesicle, young conidiophore with metulae (met), young conidiophore with phialides (ph), conidiophores with white spores (w), conidiophores with light green spores (lg), conidiophores with dark green spores (g), and young (pink) and mature (dark red, m) cleistothecia with Hülle cells. Mature conidiospores were macroscopically visible after 40 h in Hülle cells, whereas they were visible after 48 h in spores. The timing of development differs from Fig. 4 data due to different inoculation sizes.

After 24 h of growth under asexual conditions, colonies of both Hülle cells and spores showed aerial hyphae (Fig. 5a), one of the first indicators of asexual development. At the same time point, the Hülle cells featured their first young conidiophores (with white conidiospores) whereas the spores showed no clear sign of vesicle or conidiophore formation (Fig. 5). After 36 h, the number of Hülle cell-derived conidiophores with white spores had further increased and the first light green spores were present, while the spores had developed only their first conidiophores bearing white spores. Mature green spores became macroscopically visible after 40 h in Hülle cell colonies and after 48 h in spore colonies (Fig. 5). Ascospores developed initial asexual structures with a significant delay of up to 24 h in comparison to Hülle cells and conidiospores (see Fig. S2 in the supplemental material). Hülle cell-derived spores showed no apparent differences from spore-derived spores in germination or growth behavior.

10.1128/mBio.01673-20.2 FIG S1 Morphology of *sepK* deletion strain and complementation strain. The wild-type strain (wt), the Δ*sepK* mutant strain, and the complementation strain (*+ gfp-sepK*) were subjected to point inoculation with 5 × 10^3^ spores. After seven days, the colony was examined for asexual conidiospores (green) and sexual cleistothecia (c). Scale bar = 200 μm. Cleistothecia were smashed between the microscopic slide and the cover slip to detect ascospores. Scale bar = 50 μm. The Δ*sepK* deletion strain was blind to light and showed mostly sexual structures under asexual and sexual conditions. The SepK protein fused with GFP was functional after *in locus* integration into Δ*sepK*, since it complemented the *sepK* deletion phenotype. Download FIG S1, TIF file, 2.9 MB. Copyright © 2020 Troppens et al. 2020 Troppens et al. This content is distributed under the terms of the Creative Commons Attribution 4.0 International license.

10.1128/mBio.01673-20.3 FIG S2 Hülle cell germination and conidiophore formation in ascospores. (a) Representative image of 9 of a group of 20 isolated Hülle cells and evaluation of their germination status. Three cells were classified as clearly not germinated (indicated as “ng”), one as probably not germinated [indicated as “(ng)”], four as potentially germinated [indicated as “(g)”], and one as clearly germinated (indicated as “g”). (b) Asexual development is highly variable in ascospores. The most highly developed asexual structures present after 24 and 48 h under light (l) and dark (d) conditions are displayed with their frequency (1/3). No asexual structures had developed after 48 h in two of three replicates, whereas conidiophores with phialides (ph) or white spores (w) were observed in one replicate. (c) Microscopic images of germinated ascospores after 24 and 48 h. Development of a hyphal network and of asexual structures was highly variable after 48 h in both light (l) and darkness (d). Scale bar = 20 μm. Download FIG S2, TIF file, 2.1 MB. Copyright © 2020 Troppens et al. 2020 Troppens et al. This content is distributed under the terms of the Creative Commons Attribution 4.0 International license.

Grown under sexual conditions, both Hülle cells and spores developed initial asexual structures with a delay and with greater variation between the biological replicates (Fig. 5b). The times of emergence of the first early nests containing new Hülle cells were similar in the Hülle cell and spore colonies. After 3 days, Hülle cells had developed typical asexual and sexual colonies with cleistothecial precursors that matched the colonies of the spores. Similarly, after 6 days, both asexual and sexual colonies with mature cleistothecia of Hülle cells matched the spore colonies (Fig. 5b).

Taking the results together, we show here that A. nidulans possesses an as-yet-undescribed developmental program that allows accelerated conidiospore differentiation starting with multinucleate Hülle cells.

## DISCUSSION

The most prevalent biological role that was suggested for Hülle cells in the past decades was that they likely support the development of the fruiting body. This was based on the finding that Hülle cells produce enzymes that modulate the cell wall or that build up cell wall components (16–21). The idea of a contribution of Hülle cells to development was further supported by the finding that without Hülle cells, fruiting bodies still bear viable ascospores but remain significantly smaller, as exemplified by results of a study performed using the *laeA* deletion strain (22). We confirmed that Hülle cells are an active part of the hyphal network surrounding the fruiting body by demonstrating their cellular activity *in vivo*. Our analysis reveals that Hülle cells can be formed at any location along the sexual mycelium, suggesting that Hülle cells establish as a hypha-derived tissue of globular cells that could serve as a secretion platform for nourishment and protection.

Hülle cell germination had been previously suggested (15, 23–25), with the first observation dating back more than 90 years (24). However, the process itself and the subsequent fate of the cells have been poorly characterized. We provide an easy, reproducible method to induce and monitor Hülle cell germination. Our data show that, in contrast to that of spores, germination of Hülle cells is highly variable, illustrating its high sensitivity to as-yet-unknown conditions. One likely explanation of the high variability is that the vulnerable Hülle cell opening can be easily mechanically damaged, resulting in loss of cellular integrity.

Hülle cells of A. nidulans are unique in being globular fungal cells that contain several nuclei. We found that Hülle cells in a heterokaryon contain either one or, most prevalently, both of the two parental nuclei. On the basis of the different sizes and shapes that we observed in Hülle cell nuclei (Fig. 2), which are indicative of different stages of mitosis (36–39), we conclude that mitosis also occurred in attached Hülle cells, which would greatly decrease the chances for the presence of single signal nuclei and explain why these are rare.

Multinuclearity is not uncommon in fungi. The spores of some arbuscular mycorrhizal fungi, which are widely spread plant symbionts, can contain several thousand genetically diverse nuclei (40–44). It was proposed that this diversity helps the fungus to adapt to microenvironmental changes and to enhance plant growth (40, 45). It is possible that genetic diversity in Hülle cells similarly contributes to promoting the developing sexual structure in aspergilli. In the case of severe environmental disturbances, it may also offer the advantage of a short-term storage of genetic information from both parents as way of providing a form of genetic backup that lasts for the duration of ascospore development but not beyond.

In the plant and animal kingdoms, several examples of multinucleate cells that act as nurse cells have been described previously, including macrophages that promote the development of blood cells in humans (46, 47), nurse cells that surround the developing egg cell in Drosophila melanogaster (48), and endosperm cells that provide nutrients and protection to the developing embryo in flowering plants (49–51). More recently, nurse cells that provide growing oocytes with cellular material were described in mice (52). The multinucleate state in these and other systems provides a higher metabolic rate but also buffers DNA damage, which is thereby diverted from the developing structure (50, 53).

These advantages could also contribute to the accelerated spore formation that we observed in germinated detached Hülle cells, clearly giving these cells a novel biological function. In the case of a detachment, which one could speculate to have been caused by predators, Hülle cells have the potential to equip new spores with potentially useful information more rapidly than under normal conditions. Additionally, unlike spores, Hülle cells do not seem to have to swell and seem to germinate asynchronously, both of which might contribute to accelerated conidiospore development. Aspergilli can form asexual spores rather rapidly but form sexual spores only in combination with a fruiting body. Hülle cells may serve as a developmental backup for the fungus that is available under destructive conditions that may lead to the loss of the whole fruiting body.

**FIG 6:**
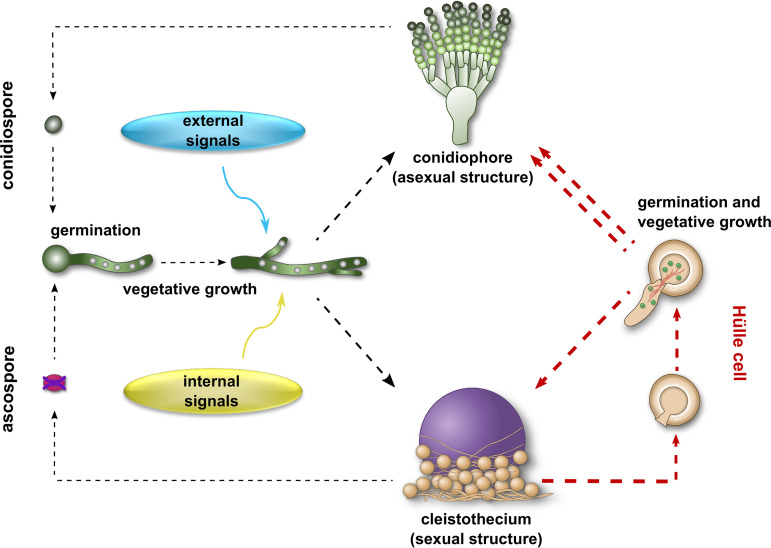
Hülle cells represent a novel branch in the life cycle of A. nidulans. Asexual spores (conidiospores) and sexual spores (ascospores) generated by the conidiophore and cleistothecium, respectively, undergo germination and vegetative growth. External and internal signals determine the entry into either asexual or sexual development. Hülle cells are mainly generated during the sexual cycle and can germinate and reinitiate both asexual and sexual development (red arrows). Hülle cells can germinate and develop asexual conidiospores more rapidly than the conidiospores themselves (illustrated by the two red arrows).

Over 5 decades ago, Hülle cells were proposed to be chlamydospores (25). Chlamydospores contain one to several nuclei and are described in several filamentous fungi and dimorphic yeasts (54–57). They develop in Candida albicans under conditions similar to those that lead to development of Hülle cells, either at terminal or intercalary positions, and can occur in chains that are similar to the patterns seen with tandem Hülle cells. Major differences occur in the cell wall, which is confined in Hülle cells but continues to the subtending hypha in *Candida* chlamydospores (57), making up less of the cell diameter (3% to 4%) (57) than is the case in Hülle cells (30%) (25). Chlamydospores do not show distinct attachment or opening sites, and germination is not restricted to certain sites, as in Hülle cells. Chlamydospore biological functions include storage or resting and long-term survival structures (54, 57). This is considerably different from Hülle cells acting as nurse cells combined with short-term rather than long-term survival and distribution functions. Hülle cells are distinct from chlamydospores and represent an as-yet-unknown fungal developmental program, giving rise to hyphae that in turn develop asexual and sexual structures and adding another level of complexity to the life cycle of A. nidulans (Fig. 6).

Hülle cells do, however, resemble stem cells, which are universally found in the plant and animal kingdom. Like stem cells, Hülle cells produce offspring cells that differentiate into specialized cells and multicellular structures, i.e., they carry the potential to differentiate into a whole new organism, making them omnipotent stem cells. Hülle cells are specialized cells (in contrast to plant and animal stem cells) that can be moved outside their niche to differentiate, similarly to stem cells (58).

## MATERIALS AND METHODS

### Strains, plasmids, and culture conditions.

All A. nidulans strains used in this study (Table 1) were cultured and maintained on minimal medium (MM) (59) and were supplemented as required (Table 1). For the auxotrophic strains, 5 mM uridine, 0.1% pyridoxine-HCl, and 5 mM uracil or 0.1% 4-aminobenzoic acid (paba) were added. For selective conditions, 100 ng/ml nourseothricin (nat), 10 μg/ml phleomycin (phleo), or 100 ng/ml pyrithiamine (ptrA) was added if required. Agar plates were incubated at 37°C and under constant white light to induce asexual development and were sealed with Parafilm and incubated in complete darkness to induce sexual development. Liquid cultures were incubated at 37°C with shaking at 120 rpm in baffled shake flasks (Schott) for vegetative growth. Conidiospores were maintained in a 0.06% NaCl–0.002% Tween suspension at 4°C.

**TABLE 1:** A. nidulans strains used in this study

Strain	Genotype	Source or reference
Wild-type A4	*veA*^+^	FGSC^*a*^
AGB551	Δ*nkuA*::*argB pyroA4 pyrG89 veA^+^*	61
AGB552	Δ*nkuA*::*argB pabaA1 veA^+^*	61
AGB506	*^P^gpdA*::*mrfp*::*h2A*/*natR pyroA4 pyrG89 veA^+^*	22
AGB990	Δ*nkuA*::*argB pyroA4 pyrG89 ^P^niiA*::*sgfp*::*h2A*::*pyrG*::*niiA^T^ veA^+^*	This study
SRS29	*pyroA4 pyrG89 ΔargB*::*trpCΔB argB^+ P^gpd*::*N-cit-1*::*gfp*	30
AGB992	Δ*nkuA*::*argB pabaA1* Δ*sepK*::*six veA^+^*	This study
AGB993	Δ*nkuA*::*argB pabaA1 gfp-sepK*::*nat*^r^ *veA^+^*	This study
AGB991	Δ*nkuA*::*argB pabaA1 gfp-sepK*::*nat*^r^ *^P^gpdA*::*mrfp*::*phleo*^r^ *veA^+^*	This study
AGB960	Δ*nkuA*::*argB pyroA4 pyrG89* Δ*dipA*::*ptrA*^r^ *veA^+^*	35

a FGSC, Fungal Genetics Stock Center.

### Crossing of A. nidulans strains.

Strains AGB506 and AGB990 were streaked crosswise on plates supplemented with required additions for both strains. The plates were incubated for asexual growth for 3 days. The mycelium was checked for areas of overlapping growth by both strains, and pieces of about 25 mm^2^ from five of these areas were cut out. Agar pieces were transferred onto selective medium containing supplements that allowed only heterokaryotic hyphae to continue to grow. The plates were incubated for sexual development for 4, 5, or 6 days. This was repeated for at least four biological replicates each with three technical replicates, two of which were quantified.

### Hülle cell growth assay.

Cleistothecia with attached Hülle cells were picked from a 5-day-old sexually grown wild-type colony, transferred to a fresh plate, rolled on the agar surface to detach individual Hülle cells, and removed. Hülle cells were incubated as indicated or were used directly for microscopy or to pick single Hülle cells.

A total of 20 Hülle cells or 20 (±1) conidiospores were picked using an MSM System 300 micromanipulator (Singer Instruments) and placed on separate fresh MM plates to compare Hülle cell and conidiospore development results. Particular care was taken to not pick Hülle cells with spores “sticking” to them. Two replicates were prepared for each time point for incubation under asexual and sexual conditions. Plates were incubated for 24, 30, 36, 40, and 48 h and for 3 and 6 days and examined. Ascospores were prepared in the same way and incubated for 24 and 48 h. An average of six biological replicates was initiated to obtain at least three valid biological replicates for asexually grown Hülle cells per time point. This was necessary due to a 50% failure rate for at least one Hülle cell among the 20 to germinate. For each time point and biological replicate, the most highly developed structure was noted. Only representative structures were used (i.e., those which occurred in at least two of three replicates) to compare the timings of asexual development in Hülle cells and conidiospores. Among the replicates, images representing the average were selected to account for variations. The rates of germination of Hülle cells and spores were quantified by microscopy at time points 24 and 30 h (and at 36 h if mycelium density allowed visual quantification). For Hülle cells, the germination rate was estimated when putative germination sites (i.e., openings) were facing downward (and were thus not clearly visible). In these cases, germination failure was assumed, but the possibility of germination could not be excluded.

### Confocal and widefield microscopy.

Five-day-old sexually grown colonies and a modified inverted agar method (60) were used to produce images of Hülle cell shapes, septation, and mitochondrial fluorescence *in vivo*. Areas with Hülle cells at the edge of the colony (lower mycelium density) and with few conidiophores of approximately 100 mm^2^ were cut out. Excessive agar was removed to increase image resolution. A drop of 0.06% NaCl–0.002% Tween solution was added into either a 2-well μ-slide (ibiTreat, Ibidi) or a 1-well chambered cover glass (Thermo Scientific), and, if required, fluorescent dye was added to the drop. FM4-64 fluorescent dye was used at a final concentration of 50 μM to stain septa, and broken or dead Hülle cells were visualized using propidium iodide at a final concentration of 150 μM. The agar piece was then inverted and placed on the drop. If propidium iodide was added, cells were incubated at room temperature for 20 min in the dark. Otherwise, cells were immediately used for microscopy.

Areas on agar plates where cleistothecia were rolled were cut out, inverted, and placed on a drop of 0.06% NaCl–0.002% Tween solution (containing fluorescent dye if required) to produce images of detached Hülle cells. For quantification purposes, at least 50 images were taken of random areas.

From areas with successful crossing, Hülle cells were removed using a toothpick and suspended in 0.06% NaCl–0.002% Tween solution. A 400-μl volume of the suspension was transferred into one well of an 8-well Lab-Tek chambered cover glass (Thermo Scientific). At least 30 images were captured that depicted at least four Hülle cells for each of the two technical replicates per time point and biological replicate.

For technical details on the microscope and software used, see Text S1 in the supplemental material.

10.1128/mBio.01673-20.1 TEXT S1 Supplemental, more-detailed description of materials and methods used in the study. Download Text S1, PDF file, 0.3 MB. Copyright © 2020 Troppens et al. 2020 Troppens et al. This content is distributed under the terms of the Creative Commons Attribution 4.0 International license.

### Transmission electron microscopy.

Hülle cells were detached as described above (Hülle cell growth assay) and grown under light conditions for 18 h to allow germination. Three to four agar pieces with grown Hülle cells in an area of about 16 mm^2^ were cut and transferred to 1.5-ml Eppendorf tubes. A solution consisting of 0.06% NaCl–0.002% Tween was added to cover the agar pieces completely. For a detailed description of sample preparation and imaging, see Text S1.

### Scanning electron microscopy.

Hülle cells were detached as described above and incubated under conditions of light exposure for 18 or 21 h. Agar pieces with germinated Hülle cells in an area of ∼2.5 cm^2^ were cut using a knife. A 2-ml screw-top reaction tube was filled with 1.5 ml agar, and Hülle cells on the agar piece were placed on the set agar in the reaction tube and held in place with a toothpick for transportation purposes. For a detailed description of sample preparation and imaging, see Text S1.

10.1128/mBio.01673-20.4 TABLE S1 Plasmids used in this study. Download Table S1, PDF file, 0.2 MB. Copyright © 2020 Troppens et al. 2020 Troppens et al. This content is distributed under the terms of the Creative Commons Attribution 4.0 International license.

10.1128/mBio.01673-20.5 TABLE S2 Primers used in this study. Download Table S2, PDF file, 0.4 MB. Copyright © 2020 Troppens et al. 2020 Troppens et al. This content is distributed under the terms of the Creative Commons Attribution 4.0 International license.
