# Supplementary figures and images for: Hülle Cells of Aspergillus nidulans with Nuclear Storage and Developmental Backup Functions Are Reminiscent of Multipotent Stem Cells
Source: mBio. 2020 Aug 11;11(4):e01673-20. doi: 10.1128/mBio.01673-20 (PMC7439468; doi:10.1128/mBio.01673-20)

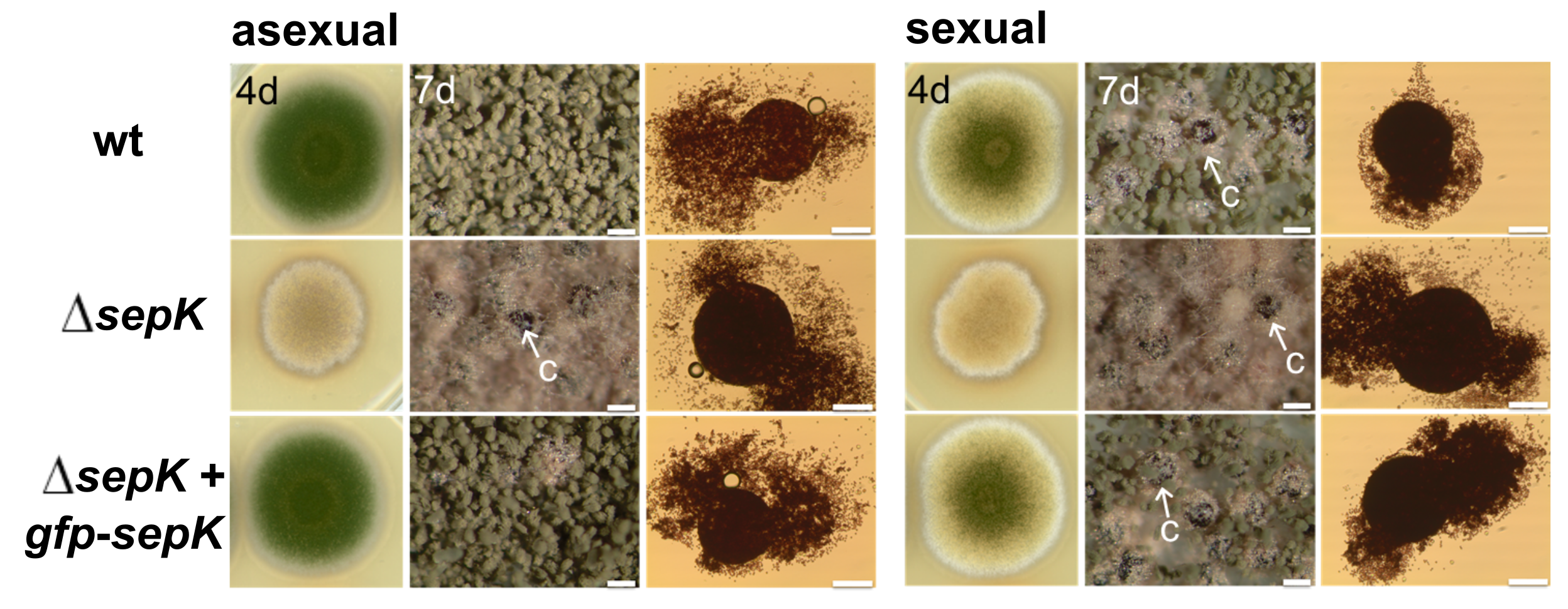

Supplement: FIG S1 [file mBio.01673-20-sf001.tif]

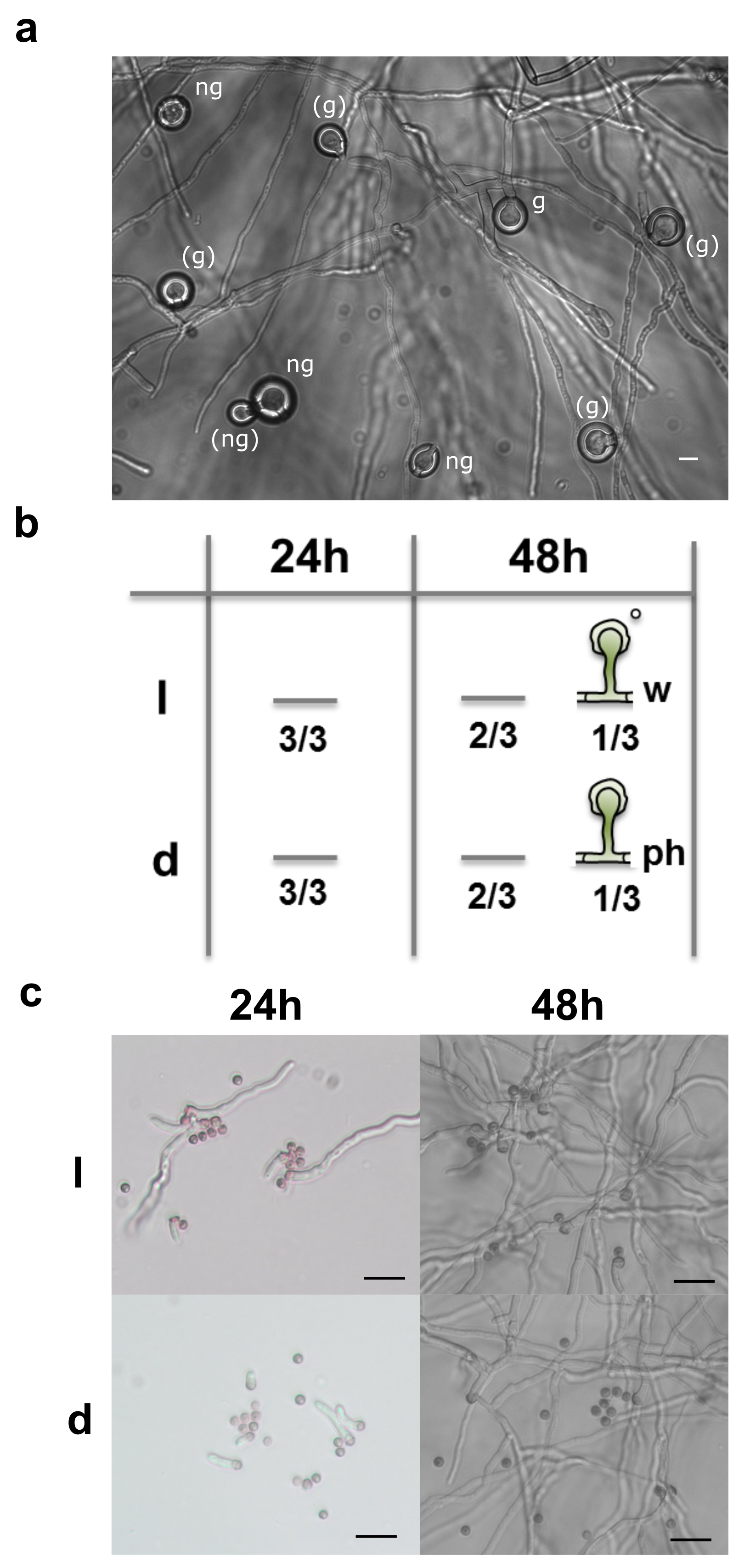

Supplement: FIG S2 [file mBio.01673-20-sf002.tif]
