# Supplementary material for: Hülle Cells of Aspergillus nidulans with Nuclear Storage and Developmental Backup Functions Are Reminiscent of Multipotent Stem Cells
Source: mBio. 2020 Aug 11;11(4):e01673-20. doi: 10.1128/mBio.01673-20 (PMC7439468; doi:10.1128/mBio.01673-20)
Supplement: TEXT S1 [file mBio.01673-20-s0001.pdf]

Supplemental Material Text S1

Hülle cells of *Aspergillus nidulans* with developmental back-up and nuclear storage functions are reminiscent of multipotent stem cells

Danielle M. Troppens, Anna M. Köhler, Rabea Schlüter[1], Michael Hoppert [2], Jennifer Gerke, and Gerhard H. Braus

Department of Molecular Microbiology and Genetics, Georg-August-University Göttingen, Germany, [1] Imaging Center of the Department of Biology, University of Greifswald, Germany, [2] Department of General Microbiology Georg-August-University Göttingen, Germany

Plasmid and strain construction

Plasmids (Supplementary table S1) and primers (Supplementary table S2) were designed using the DNASTAR software SeqBuilder 12.1.0. The *sepK* deletion strain was generated by replacing the *sepK* gene with a *nourseothricin (nat)*-flipper resistance marker cassette (1). Wildtype genomic DNA was used as template for amplification of *sepK* 5' flanking region using primer AL01/AL02 and AL03/AL04 for the 3' flanking region. pME4304 containing the *nat-flipper* resistance marker cassette was digested with *Sfi*I. The 5', 3' flanking regions and *nat* cassette were ligated to linear pUC19 resulting in plasmid pME4539. For complementing Δ*sepK, gfp* was fused N-terminally to the genomic sequence. The *sepK* 5' flanking region was amplified from wildtype genomic DNA using AL24/AL25. The *gfp* including a linker region and precision protease cleavage site was amplified with EB10/oAMK95. The *sepK* gene was amplified using AL26/AL27. All three fragments were ligated to linear pUC19. The plasmid was linearized with *Swa*I. A second ligation step was performed to insert a non-recyclable *nat* marker cassette amplified with AL28/oAMK80 and the *sepK* 3'

flanking region amplified with AL29/AL30. The resulting plasmid was named pME4540. To visualize nuclei, *gfp* was N-terminally fused to the histone *h2A* gene under the nitrate inducible *niiA* promoter and using a *pyrG* marker for selection (pME4541). For this, *gfp-h2A* was amplified from pME4542 using primers DT60/DT61 with overlaps to pME3160 on both ends. Plasmids were assembled using the seamless cloning and assembly mix (Invitrogen). The cloning mix was transformed into competent cells of *E. coli* DH5α or DH10B that were selectively grown on Lysogeny Broth (1% tryptone, 0.5% yeast extract, 0.5% NaCl) medium supplemented with 100 µg/ml ampicillin. Plasmids were checked for errors in base pair sequences by Sanger Sequencing (Sequence Laboratories Göttingen) and only used further if error-free. Plasmids containing *h2A* fused to *gfp* (pME4541) and *rfp* (pME3857) were used directly for random ectopic integration into AGB551 and AGB993, respectively, leaving the native *h2a* gene intact. Promoters of *gpdA* and *niiA* were used for constitutive or constantly induced expression of RFP and GFP, respectively. Plasmids designed for gene knockout (pME4539) or complementation (pME4540) were digested with *Pme*I before integrating them *in locus* for native expression into AGB552 (to obtain AGB992) and AGB992 (to obtain AGB991), respectively.

A. nidulans transformation was performed as previously described with modifications (2). In brief, mycelium of an overnight culture was harvested and protoplastation was achieved by using an enzyme mixture of VinoTaste (Novozymes) and lysozyme (Serva). A minimum of 1.2 µg of digested or undigested plasmid DNA was added to the protoplasts. Transformation mixture was plated on selective medium containing 1.2 M sorbitol for protoplast maintenance. The correct integration was verified by Southern hybridization using the Amersham AlkPhos Direct Labelling and Detection System (GE Healthcare) according to manufacturer’s instruction.

Genomic DNA was extracted from liquid overnight cultures as described before (2) with the modification that phenol/chloroform was replaced with potassium acetate and isopropanol. Genomic DNAs of positive transformants and parent strains were digested overnight with appropriate restriction enzyme to obtain differently sized fragments for distinction of modified and wildtype strains. The probes were designed to bind to the flanking region of the gene of interest that was part of the plasmid construct. After verification by Southern hybridization the recyclable *nat* marker was removed by ß-recombinase activity induced by growing the strain on 1% xylose medium. Successful complementation of the deletion phenotype of ∆sepK (AGB992) by GFP-SepK (AGB993) was confirmed by plating both strains on minimal medium to compare with wildtype growth (Supplemental Material Figure S1).

Confocal microscopy

Microscopic chambers were transferred to an inverted Zeiss Axio Observer Z.1 microscope coupled to a confocal Yokogawa CSU-X1 spinning disc. Images were captured using a Zeiss PlanAPOCHROMAT 63x/Air objective or a ZEISS PlanAPOCHROMAT 20x/Air objective, a QuantEM:512SC (Photometrics) for confocal fluorescence or a Coolsnap HQ^2 (Photometrics) camera for widefield differential interference contrast. The SlideBook 6.0 imaging software (Intelligent Imaging Innovations Inc.) was used.

For macroscopic images, either an Olympus SZX12 stereo microscope with the cellSens Dimensions 1.4 software (Olympus) or an Epson Perfection V600 Photo scanner with the Epson Scan software was used.

Transmission electron microscopy

For fixation, 2.5% (v/v, final concentration) glutaraldehyde was added. The sample was

incubated on ice for 90 minutes and then dehydrated by stepwise addition and complete removal of 15%, 30%, 50%, 70%, 95% and 100% ethanol (Ethanol absolute, VWR Chemicals) on ice for 15 (only for 15%) or 30 minutes, respectively. The step with 100% ethanol was repeated once. For a stepwise removal of ethanol and for embedding, a 33.3% ethanol/66.6% LR White resin (Plano GmbH, Wetzlar) mixture was added to the sample and incubated for two hours. After complete removal of this mixture, 100% LR White resin was added and incubated overnight at 4°C. The sample pieces were individually placed upside down into a gelatine capsule (Plano GmbH, Wetzlar) and fresh LR White resin was added. Polymerization of the resin was achieved by incubation of the capsule in a 55°C oven for at least 16 hours. Before preparing ultra-thin sections (80 nm) of the sample using a Reichert Ultracut E ultramicrotome (Leica Microsysteme GmbH, Wetzlar), excessive resin was removed using a milling tool (TM 60, Reichert, Vienna, Austria). Sections were manually placed onto Formvar-coated copper grids (300 mesh, Plano GmbH, Wetzlar), dried, stained with uranyl acetyl (4%, w/v) for five minutes and transferred to a Jeol JEM1011 transmission electron microscope (Jeol GmbH, Eching). Images were captured using a Gatan Orius 4 K camera and processed with the Gatan Digital Micrograph software package (Gatan, GmbH, Munich).

Scanning electron microscopy

After a fixation step (2.5% glutaraldehyde, 2% paraformaldehyde, 1 mM $CaCl_2$, 1 mM $MgCl_2$, 0.5% Tween 20, 50 mM NaN_3 in 100 mM HEPES [pH 7.5]), samples were treated with amino acid-sucrose-solution (2% arginine, 2% glycine, 2% glutamate, 2% sucrose) microwaved for 5 min at 30 °C, guanidine-tannin-solution (2% guanidine, 2% tannin) microwaved for 5 minutes at 30 °C, and 1% osmium tetroxide in 100 mM cacodylate buffer (pH 7; supplemented with 1 mM $CaCl_2$, 25 mM NaN_3) for 30 min at

room temperature with washing steps in between. The samples were dehydrated in a graded series of aqueous ethanol solutions (10 – 100%) and then critical point-dried. Finally, samples were mounted on aluminum stubs sputtered with gold/palladium and examined with a scanning electron microscope EVO LS10 (Carl Zeiss Microscopy GmbH, Oberkochen, Germany).

Computational analysis

Microscopic images were edited and analyzed with the image processing software ImageJ (3). The cellcounter plugin (Kurt De Vos, http://imagej.net/Cell_Counter) was used to quantify Hülle cell shapes. Hülle cells were considered terminal when either other Hülle cells or hyphal branches diverged from their subtending hypha. In all other cases, they were considered intercalary. Co-localization of fluorescent nuclei was analyzed using a customized version of the colocalization plugin (Pierre Bourdoncle, Institut Jacques Monod, Service Imagerie, Paris, http://rsb.info.nih.gov/ij/plugins/index.html#analysis). Only Hülle cells containing nuclei distinctly recognizable as such were counted, whereas Hülle cells displaying undefined fluorescence were discarded. Only biological replicates with at least one technical replicate giving a minimum of 100 valid Hülle cells were taken into account to calculate ratios of nuclei variants. If both technical replicates were valid, the average was used.

DNA sequences for plasmid construction were retrieved from AspGD (http://www.aspgd.org), edited with Seqbuilder 12.1.0 (DNAStar Lasergene), and sequence analysis and DNA alignments were performed using ApE (http://biologylabs.utah.edu/jorgensen/wayned/ape/). Statistical analysis (as indicated) was performed in R ((4); http://www.R-project.org) or in Microsoft Excel.

References:

1. Thieme KG, Gerke J, Sasse C, Valerius O, Thieme S, Karimi R, Heinrich AK, Finkernagel F, Smith K, Bode HB, Freitag M, Ram AFJ, Braus GH. 2018. Velvet domain protein VosA represses the zinc cluster transcription factor SclB regulatory network for *Aspergillus nidulans* asexual development, oxidative stress response and secondary metabolism. PLoS Genet.
2. Punt P, van den Hondel C. 1992. Transformation of filamentous fungi based on hygromycin B and phleomycin resistance markers. Methods Enzymol 216:447–457.
3. Schneider C, Rasband W, Eliceiri K. 2012. NIH Image to ImageJ: 25 years of image analysis. Nat Methods 9:671–675.
4. Team RDC. 2008. R: A language and environment for statistical computing. R Foundation for Statistical Computing, Vienna, Austria.
