# Supplementary material for: Hülle Cells of Aspergillus nidulans with Nuclear Storage and Developmental Backup Functions Are Reminiscent of Multipotent Stem Cells
Source: mBio. 2020 Aug 11;11(4):e01673-20. doi: 10.1128/mBio.01673-20 (PMC7439468; doi:10.1128/mBio.01673-20)
Supplement: TABLE S1 [file mBio.01673-20-st001.pdf]

Table S1: Plasmids used in this study.

Plasmid	Description	Source/Reference
pBluescript II KS+	cloning plasmid	Stratagene
pUC19	cloning plasmid	Invitrogen
pAN8-1	phleomycin resistance cloning plasmid	(1)
pME3160	*niiA/niiD* expression module with *pyrG* marker	(2)
pME3857	^{P}*gpdA*::*mRFP*::*h2A* in pAN8-1	(3)
pME4304	*recyclable nat marker*	(4)
pME4539	^{P}*sepK*::*nat*^{R}::*sepK*^{t} deletion in pUC19	This study
pME4540	^{P}*sepK::sgfp*::*sepK*::*nat*^{R} in pUC19	This study
pME4541	^{P}*niiA::sgfp::h2A::pyrG::niaD*^{T} in pME3160	This study
pME4542	^{P}*h2A::sgfp::h2A::phleo*^{R} in pBluescript II KS+	This study

References:

1. Punt P, van den Hondel C. 1992. Transformation of filamentous fungi based on hygromycin B and phleomycin resistance markers. Methods Enzymol 216:447–457.

2. Bayram O, Krappmann S, Ni M, Bok JW, Helmstaedt K, Valerius O, Braus-Stromeyer S, Kwon N-J, Keller NP, Yu J-H, Braus GH. 2008. VelB/VeA/LaeA Complex Coordinates Light Signal with Fungal Development and Secondary Metabolism. Science (80-) 320:1504–1506.

3. Bayram Ö, Bayram ÖS, Ahmed YL, Maruyama J, Valerius O, Rizzoli SO,

Ficner R, Irniger S, Braus GH. 2012. The *Aspergillus nidulans* MAPK Module AnSte11-Ste50- Ste7-Fus3 Controls Development and Secondary Metabolism. PLoS Genet 8.

4. Thieme KG, Gerke J, Sasse C, Valerius O, Thieme S, Karimi R, Heinrich AK, Finkernagel F, Smith K, Bode HB, Freitag M, Ram AFJ, Braus GH. 2018. Velvet domain protein VosA represses the zinc cluster transcription factor SclB regulatory network for *Aspergillus nidulans* asexual development, oxidative stress response and secondary metabolism. PLoS Genet.
