# Supplementary material for: Hülle Cells of Aspergillus nidulans with Nuclear Storage and Developmental Backup Functions Are Reminiscent of Multipotent Stem Cells
Source: mBio. 2020 Aug 11;11(4):e01673-20. doi: 10.1128/mBio.01673-20 (PMC7439468; doi:10.1128/mBio.01673-20)
Supplement: TABLE S2 [file mBio.01673-20-st002.pdf]

Table S2: Primer used in this study.

Primer	5'-3' Sequence (number of nucleotides)	Target
AL01	cctataggcctgagtTCATTTGATTCTTTTGATTACCCC (39)	5' *sepK*
AL02	attcgagctcggtacGTTTAAACCTCTCACTGACACTGAAGC (42)	5' *sepK*
AL03	ataatatggccatctTGTCTGAGTATGAGAAGCAATCC (38)	3' *sepK*
AL04	ccaagcttgcatgccGTTTAAACGCGCCGACTCGGGAT (38)	3' *sepK*
AL24	attcgagctcggtacGTTTAAACCACTTAACGCGCCGAC (39)	5' *sepK*
AL25	GCCCTTGCTCACCATTGTCTGAGTATGAGAAGCAATCC (38)	5' *sepK*
AL26	GGTGGTAGCGGTGGTATGAGTAATCAGCCATGGCTG (36)	*sepK*
AL27	ccaagcttgcatgccATTTAAATTCAACCGGTGATCGCTTTC (42)	*sepK*
AL28	TCACCGGTTGAATTTGGCGGCTCTGAGGTGCAGT (34)	*nat*
AL29	ATGCCCTGCCCCTGATCATTTGATTCTTTTGATTACCCCTT (41)	3' *sepK*
AL30	ccaagcttgcatgccGTTTAAACAACAGAGCGCTTACGAA (40)	3' *sepK*

EB10	ATGGTGAGCAAGGGCGAGG (19)	*sgfp*
oAMK80	TCAGGGGCAGGGCATGCTC (19)	*nat*
oAMK95	ACCACCGCTACCACCggg (18)	*sgfp*
DT60	CAT ACT CTC ACA TTT ATG GTG AGC AAG GGC GAG GAG (36)	s*gfp*
DT61	TGC GAACCC GTA TTT TTA CAG CTC CTG GCT GCC CTT (36)	*h2A*
